# Researching, co-creating and testing innovations in paper-based health information systems (PHISICC) to support health workers’ decision-making: protocol of a multi-country, transdisciplinary, mixed-methods research programme in three sub-Saharan countries

**DOI:** 10.1186/s12961-021-00768-0

**Published:** 2021-08-11

**Authors:** Xavier Bosch-Capblanch, David O’Donnell, L. Kendall Krause, Christian Auer, Angela Oyo-Ita, Mamadou Samba, Graça Matsinhe, Abdullahi Bulama Garba, Damaris Rodríguez, Meike Zuske, Anthonia Ngozi Njepuome, Sofia Micael Mandjate Lee, Amanda Ross, Suzanne Gajewski, Artur Manuel Muloliwa, Richard B. Yapi, David W. Brown

**Affiliations:** 1 Swiss Tropical and Public Health Institute, Basel, Switzerland; 2 University of Basel, Basel, Switzerland; 3 Post Normal PLC, Chicago, IL United States of America; 4 Bill & Melinda Gates Foundation, Seattle, WA United States of America; 5 Department of Community Medicine, University of Calabar, Calabar, Nigeria; 6 Ministère de La Santé et de l’Hygiène Publique, Abidjan, Côte d’Ivoire; 7 Université Félix Houphouet Boigny, Abidjan, Côte d’Ivoire; 8 Expanded Program on Immunization, Ministry of Health, Maputo, Mozambique; 9 Director Planning, Research and Statistics, National Primary Health Care Development Agency, Abuja, Nigeria; 10 Sonder Collective, Helsinki, Finland; 11 Swiss Tropical and Public Health Institute, Abuja, Nigeria; 12 Swiss Tropical and Public Health Institute, Manhiça, Mozambique; 13 Faculty of Health Sciences, Lúrio University, Nampula, Mozambique; 14 Centre Suisse de Recherches Scientifiques en Côte d’Ivoire, Abidjan, Côte d’Ivoire; 15 Centre d’Entomologie Médicale et Vétérinaire, Université Alassane Ouattara, Bouaké, Côte d’Ivoire; 16 BCGI LLC / pivot-23.5°, Cornelius, NC United States of America

**Keywords:** Primary healthcare, Decision-making, Health information system, Human-centred design, Sub-Saharan Africa, Côte d’Ivoire, Mozambique, Nigeria

## Abstract

**Background:**

Health information systems are crucial to provide data for decision-making and demand for data is constantly growing. However, the link between data and decisions is not always rational or linear and the management of data ends up overloading frontline health workers, which may compromise quality of healthcare delivery. Despite limited evidence, there is an increasing push for the digitalization of health information systems, which poses enormous challenges, particularly in remote, rural settings in low- and middle-income countries. Paper-based tools will continue to be used in combination with digital solutions and this calls for efforts to make them more responsive to local needs. Paper-based Health Information Systems in Comprehensive Care (PHISICC) is a transdisciplinary, multi-country research initiative to create and test innovative paper-based health information systems in three sub-Saharan African countries.

**Methods/Design:**

The PHISICC initiative is being carried out in remote, rural settings in Côte d’Ivoire, Mozambique and Nigeria through partnership with ministries of health and research institutions. We began with research syntheses to acquire the most up-to-date knowledge on health information systems. These were coupled with fieldwork in the three countries to understand the current design, patterns and contexts of use, and healthcare worker perspectives. Frontline health workers, with designers and researchers, used co-creation methods to produce the new PHISICC tools. This suite of tools is being tested in the three countries in three cluster-randomized controlled trials. Throughout the project, we have engaged with a wide range of stakeholders and have maintained the highest scientific standards to ensure that results are relevant to the realities in the three countries.

**Discussion:**

We have deployed a comprehensive research approach to ensure the robustness and future policy uptake of findings. Besides the innovative PHISICC paper-based tools, our process is in itself innovative. Rather than emphasizing the technical dimensions of data management, we focused instead on frontline health workers’ data use and decision-making. By tackling the whole scope of primary healthcare areas rather than a subset of them, we have developed an entirely new design and visual language for a suite of tools across healthcare areas. The initiative is being tested in remote, rural areas where the most vulnerable live.

## Background

Better decisions in the clinical, public health or policy domains in healthcare require high-quality evidence on the problems tackled and on potential solutions and their implications [1]. Decision-making is operationalized through health information systems (HIS), which encompass data sources and processes that are meant to inform decisions across the whole healthcare system [2]. HIS include subsystems such as clinical records, disease surveillance, routine health management information systems (HMIS) and logistics management information systems, as well as financial data [3]. However, HMIS can fail to respond comprehensively to data demands for decisions; in particular, information systems may not be used by decision-makers. This is an issue which has been a matter of debate for decades [4], as exemplified by the data-related issues raised during the current COVID-19 pandemic [5, 6].

Linkages between information and decisions are complex, poorly understood and inconsistent, and do not necessarily follow transparent and systematic processes [7]. Even with imperfect information, reasonable decisions can be (and are) made [8], yet research evidence suggests that data and decisions are often disconnected, with examples from several managerial levels of the health systems [9, 10]. To aggravate this problem, evidence on HMIS interventions is patchy and largely inconclusive [4].

Furthermore, accountability requirements at the international [11] and national levels [12] have increased the demand for more and better data from countries and eventually frontline civil servants. For example, the Sustainable Development Goals have a set of 247 indicators across 17 goals [13].

Frontline health workers are the primary data collectors at the periphery of the system. They collect health data during the provision of health services and aggregate and transmit them to the higher tiers of the system. They face the challenge of responding to increasing data demands from managers and stakeholders while having to provide good-quality care [14] in their daily routine work. While complaints about data quality have been reported for long time [15], substantial efforts have been made to systematize HIS [16], measuring and demanding better data quality [17]. These demands have been addressed through some promising digital interventions [18]; however, their design and implementation have shown important caveats [49], particularly in low- and middle-income countries (LMIC), where the need for reliable electrical supply, connectivity, equipment and funding impede their implementation. Moreover, research evidence on the effects of digital solutions is also patchy and inconsistent, even in high-income countries settings, where issues around digitalization in healthcare have been raised [19, 20]. Hence, it is very likely that paper tools will remain a substantial—when not the only—data support technology, particularly in remote, rural health facilities in many countries. Yet, little research exists on how frontline health workers interact with existing paper tools, and even less is understood about how these tools can be redesigned to improve data quality and use.

The Bill & Melinda Gates Foundation (BMGF) recognized the opportunity to invest in research on paper-based tools, issuing a call titled “Operations Research on Improving Paper-based Information Systems for Child Health”, with the aim of designing an innovative paper-based information system for primary healthcare (PHC) and testing the system’s effectiveness with respect to data quality and the provision of care. We describe herein the overall PHISICC (Paper-based Health Information System in Comprehensive Care) research programme approach. PHISICC is a multi-year, multi-country, transdisciplinary, mixed-methods research project aimed at producing and testing an innovative paper-based HIS in three sub-Saharan African countries.

## Methods/Design

### Aims

The main aims of PHISICC are to create an innovative HIS for PHC and to assess its effectiveness with regard to data use and quality, quality of healthcare and healthcare worker perceptions about the new tools, applied in rural settings in Côte d’Ivoire, Mozambique and Nigeria. The project was approved at the end of 2015, and research activities are planned till mid-2021.

Specifically, we aim at addressing the following research components, organized into six workstreams (WS):

1. Setting up partnerships between research institutions and the governmental health sector in the three countries, to ensure country ownership, political relevance and scientific excellence (WS1)
2. Synthesizing the research literature on the effects of HIS and carrying out a framework synthesis to understand how HIS are portrayed in the research literature (WS2)
3. Characterizing the use of HIS in the daily practice of frontline health workers in Côte d’Ivoire, Mozambique and Nigeria (WS3)
4. Designing an innovative paper-based HIS for the three countries (i.e. PHISICC paper-based tools) using co-creation processes, human-centred design and services design (WS4)
5. Assessing the effects of the PHISICC tools on data quality, data use and quality of care, through experimental studies in the three countries (WS5)
6. Disseminating the findings and advocating for policy uptake of the new evidence produced (WS6).

### Overall research setup and rationale

The project proposal was developed by a core team composed of the main applicant institution, the design partner and countries’ researchers. As the proposal was being drafted, governmental health sector officials from LMIC in Africa and Asia were contacted to ascertain their participation interest for a research project focusing on paper-based HIS. Contacts were made leveraging past and current partnerships of the Swiss Tropical and Public Health Institute (Swiss TPH). We established five criteria to engage with a country: (i) the governmental health sector considered that the paper components of the HMIS were going to remain for a long time period; (ii) they acknowledge the existence of data quality issues; (iii) they demonstrated that data quality and use was a strategic priority; (iv) they were willing to accept transient changes to the HIS for research purposes; and (v) they considered the research relevant to eventually feed into future policy or strategic planning exercises.

**Fig. 1:**
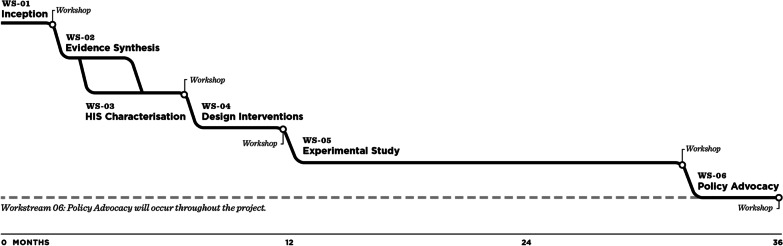
PHISICC programme timeline

The research project revolves around the creation of a new paper-based HIS intervention, using a design process employing co-design, human-centred design (HCD) and service design (WS4) approaches, and testing it using experimental study designs (WS5). It was critical that the design process be informed by (i) existing evidence, (ii) real practices and (iii) workers’ HCD principles. For these reasons, the design work (WS4) was preceded by a systematic review of the literature (WS2) and characterization of the HIS in the countries (WS3). The findings from WS2 and WS3 were synthesized and entered into the co-creation design process (WS4). Equally important was the need to keep the whole research grounded on the real-life settings of the countries and relevant to the strategic priorities of the ministries of health. Hence, we established a WS6 to ensure the engagement, communication and advocacy with partners from the very conception of the project with concrete activities. The initial timeline of the project is shown in Fig. 1.

### WS1: Management and inception

The aims of the inception phase were to set up administrative and managerial procedures, formalize partnerships and draft generic protocols for each WS. In each country, we established two main engagements: we signed a memorandum of understanding with the relevant department of the governmental health sector and we entered into contractual arrangements with partner national research institutions. Research partners were selected applying the following criteria: (i) they had, or were interested in having, a portfolio on health systems research; (ii) they had the capacity to manage a long-term project within a wider country partnership; and (iii) the PHISICC project was consistent with their own research priorities.

In parallel, we identified international experts who could act as members of a technical advisory group (TAG). The main function of the TAG is to support project partners in project design and implementation, and to monitor progress. We searched and selected experts on HIS, a public healthcare area (e.g. immunization), research synthesis and policy, and HCD. TAG members operate on a voluntary basis. A chair was designated by consensus among the six TAG members, and two representatives of the funding agency joined the TAG as observers. In the first year of the project, there was one “global” workshop including the project team with country partners and the TAG.

### WS2: Global evidence synthesis

We carried out a systematic literature review in order to synthesize the evidence on the effects of interventions targeting HIS [21, 22], following standardized and widely accepted methods [23, 24] and reporting practices. We included studies where participants were healthcare providers or managers of health services. The interventions of interest had to target HIS and report on data quality and use or decision-making. We searched the Cochrane Central Register of Controlled Trials (CENTRAL), Ovid MEDLINE and Ovid MEDLINE In-Process & Other Non-Indexed Citations, EMBASE, Epistemonikos, Health Systems Evidence, PDQ-Evidence, SUPPORT Summaries, the Grey Literature Report, OpenGrey and the WHO Global Health Library, as well as websites and trial registries. The search strategy was adapted to each literature database. To address this “effectiveness” question, we included experimental and quasi-experimental studies [25], in any language, carried out in LMIC. We included studies of any type of intervention targeting HIS because we could not assume that studies on, for instance, digital HIS interventions would not have findings or lessons relevant to paper tools. We did not restrict the concept of HIS to the routine HMIS, because we wanted to capture other sources of information that may come into play when making decisions, such as survey results, evaluations or even anecdotal evidence.

While the systematic review was meant to provide evidence on the effects of interventions, the TAG raised concerns that in the systematic review on effectiveness we would miss evidence on systems, processes and human experiences with the information systems. The TAG encouraged the team to carry out another research synthesis to provide additional evidence on the characteristics of the HIS and its use for decision-making, as reported in the research literature. Following this recommendation, we carried out a systematic “framework synthesis” [26, 27] based on observational studies, both quantitative and qualitative. The participants and scope of the research synthesis were the same as for the effectiveness systematic review, except that we focused on observational and qualitative studies.

### WS3: Local evidence on HIS

We complemented the global evidence gathered in WS2 with local evidence through a characterization of the HIS in their context of use. The aim of this WS was to acquire a deep understating of the HIS tools, structure and functioning in the real setup of rural health facilities, in order to inform the intervention design (WS4). Participants in these studies included frontline health workers in Côte d’Ivoire, Mozambique and Nigeria from purposively selected health facilities. Since our focus was to analyse and understand HIS processes, we did not attempt to achieve a large or representative sample of health facilities. These characterizations were carried out in 2-week field visits to remote, rural areas in each country, with a team of researchers, HCD experts and governmental health sector officials [28].

Visits were guided by a protocol and were structured around four main activities: an initial orientation workshop, in-depth interviews with stakeholders, visits to health facilities and feedback workshops. In the initial workshop, partners and stakeholders were updated on project progress and the field visit protocol was shared and adapted. Participatory techniques were used to facilitate engagement and to gather feedback. In-depth interviews addressed the roles of the main stakeholders in identifying and addressing HIS challenges.

Field visits were focused on (i) describing the general features of health facilities and the HIS (ii) understanding the experience of frontline health workers using the HIS and (iii) assessing the data quality of selected indicators in the HIS. A novel feature of the PHISICC approach was the application of HCD to understand the frontline health workers’ experience with the HIS. The HCD approach involved 3 to 5 hours of observations and interviews at each health facility. Activities included shadowing health workers to observe their work with patients and tools, and interviewing them to understand their work, their challenges and the degree to which current HIS tools supported or undermined their ability to do that work. Site visits and interviews were videotaped for detailed review and analysis. Current HIS tools and paper-based registers were photographed to create a visual inventory of HIS in context. Informed consent was obtained from health workers and patients prior to recording, and individualized patient information was scrubbed.

### WS4: Designing an innovative paper-based HIS using a design process

The aim of WS4 was to create and produce a set of innovative and improved paper-based tools for routine use in the HIS in the three countries. The performance of these tools is to be tested in WS5. The design process consisted of co-design and service design methods with a HCD approach. HCD has been widely used to ascertain users’ points of view while at the same time developing solutions to tackle critical user problems. It has been used in both the public [29] and private [30] sector organizational context.

Design has been used as a creative problem-solving approach to translate the findings from WS2 and WS3 into usable, desirable, feasible and effective tools [31]. A HCD approach meant that the lived experiences of the HIS users, namely healthcare providers, would be a key aspect of the subsequent design decisions (i.e. desirability and usability) around clinical standards and guidelines (e.g. integrated management of childhood illness [32]).

Co-design methods are processes that involve both the designers' creative activity and people not trained in design, working together in the design development process. Service design methods involve activities for thinking through the HIS conceptualization as a service with providers and end-users, using tools such as journey mapping, prototyping and information design to achieve consistent and seamless service experiences [33]. Co-creation groups were formed to enable the co-design activities, with 12 members from the three partner countries. The co-creation team's role was to produce and select the tools versions, providing their in-country experience, technical expertise and contextual understanding. The group also contributed to user testing activities with frontline workers to iterate the tools based on user feedback [34]. Therefore, participants in the co-creation processes included frontline health workers, managers familiar with HIS and the HCD designers as well.

The new PHISICC paper tools include all major healthcare areas in PHC, namely antenatal care, delivery, postnatal care, vaccination, sick child, outpatient consultations, HIV and tuberculosis, and the recording, tallying and reporting subsystems (i.e. recording with register books for individual patient care and tallying and reporting to provide aggregated figures to the higher tiers of the health system). Tools were harmonized across countries, produced in their official languages (English in Nigeria, French in Côte d’Ivoire and Portuguese in Mozambique) and adapted to country-specific requirements (e.g. different vaccination schedules). The final versions of the tools were locally produced to supply health facilities in the intervention arms during the testing period (see WS5).

### WS5: Evaluation of the intervention using randomized controlled trials

The aim of WS5 was to test the effectiveness of the innovative PHISICC paper-based tools with respect to data quality and use. The PHISICC tools are being tested in real-life situations in the three countries, using a cluster randomized controlled trial design. Details on the trial protocol are available elsewhere, including its registry [35, 36]. Briefly, study areas were selected based on the availability of remote, rural health facilities, serving vulnerable populations, and being reasonably accessible to carry out research activities. We obtained a master list of health facilities corresponding to the study areas from governmental health authorities. We selected health facilities (the unit of intervention) and households (to estimate community-based outcomes). Health facilities were randomly allocated to the intervention or control arms by blindly pulling equal-sized pieces of paper with the names of the health facilities on them, one by one, from an opaque bag. The sample size required was calculated for each primary outcome with a power of 80%: in each country, 35 health facilities were allocated to each arm. In the intervention arm, current HIS paper-based tools were replaced with the new PHISICC tools in order to avoid duplicate recording and reporting. In each health facility catchment area we randomly selected three villages and 10 households per village to estimate community-level outcomes.

Outcomes will be assessed at the health facility and household level. The outcomes of interest include (i) health-related outcomes (e.g. percentage of vaccinated children); (ii) data quality (e.g. ratio of recorded and reported healthcare events) and data use outcomes (e.g. average number of diagnoses and rate of correct treatments); (iii) user-related outcomes, for both service providers and patients (e.g. acceptability and usability of new tools); and (iv) resource consumption outcomes (e.g. time spent in reporting data, costs). The effectiveness of the intervention will be estimated by comparing intervention and control arms at end-line (for outcomes where only the end-line is available) or differences between baseline and end-line (for outcomes where both baseline and end-line are measured) using regression models, taking the structure of the data into account (e.g. logistic regression will be used for binary outcomes, and Poisson regression for data quality, depending on the observed distributions).

Understanding that the RCT would provide evidence on the effectiveness of the intervention, but not necessarily on how and why it may or may not have succeeded, we are gathering qualitative data using a HCD approach as well. The main issues to address include how frontline health workers interact with the tools and their views on design issues that require modification or improvement (health facility laboratories).

### WS6: Stakeholder engagement, communication and policy advocacy

The aim of WS6 is to keep the whole PHISICC research programme relevant to the real situation of HIS in the three countries, as well as allowing partners and stakeholders to participate in the research design and in the interpretation of findings. WS6 started at the very inception of the project and remains active along the whole project activities (Fig. 1). Participants include research partners, frontline health workers, health services managers and stakeholders, who have been engaged along the programme. Stakeholder engagement and country ownership are core components of this research to keep it rooted in the reality of the three countries and relevant to the policy contexts. To this end, we have actively adhered to the 11 principles of transboundary research [37], as shown in Table 1.

**Table 1:** Principles of transboundary research partnership

	Principle	How PHISICC has implemented the principle	Examples of results
1	Set the agenda together	Formal partnership between the Swiss TPH, national research institutions and ministries of health; participation since before proposal submission	Agreed protocols for each WS
2	Interact with stakeholders	Stakeholder consultations through workshops and other exchanges	Stakeholder analyses; incorporation of stakeholder views
3	Clarify responsibilities	Establishment of generic research management structure and then adapted to each country	Each WS is “governed” by specific terms of reference, which are agreed to among partners
4	Account to beneficiaries	Transparent and systematic exchanges between funding agency, management and implementation	Monthly calls with the BMGF; trial monitoring; reporting to directorates of involved institutions
5	Promote mutual learning	Uncountable forums where issues have been discussed across the whole team, including senior and junior researchers and partners	PHISICC has gradually incorporated dimensions of human resources and quality of care to the initial focus of the project on HIS
6	Enhance capacities	Fluid communication about global health research and funding opportunities	Collaborations with partners already extend beyond PHISICC
7	Share data and networks	Transparent data management mechanisms; systematic approach to evidence production and use	Project newsletter; shared databases; internal memos in critical stages of the project
8	Disseminate results	PHISICC dissemination and advocacy plan produced	PHISICC preliminary findings for presentation in conferences; manuscripts sent to peer-reviewed journals; discussions with partners for policy uptake
9	Pool profits and merits	Active promotion of partners’ visibility in events and initiatives	Copyright of PHISICC tools attributed to the project partnership
10	Apply results	Policy uptake plans being produced	Conversations on policy uptake already started
11	Secure outcomes	Post-PHISICC plans being produced in terms of (i) further applications of HCD and public health collaborations, (ii) applying lessons learned to other subcomponents of the HIS and (iii) considerations on scaling up and sustainability	No tangible results yet

Communication tools are being leveraged to increase awareness of PHISICC and to allow stakeholders to follow up on events. These include an activity report [38], a website (phisicc.org), a Twitter account (@phisicc_), a newsletter (“The Tally”) and numerous internal memos and updates.

WS6 includes plans to take up the evidence and lessons learned from PHISICC, which go far beyond the findings of the WS5, and include approaches, methods and lessons learned from interactions with frontline health workers.

### Status of the study

In all three countries, WS3 and WS4 have come to an end. Currently the trial (WS5) is ongoing in each of the three countries. Data collection has begun in the sense that the baseline data collection was completed in 2020. At the time of the manuscript publication, data collection had ended in the three countries.

## Discussion

Research on health systems is challenging due to the complexity of the systems, the limited knowledge of their functioning, the influence of contextual issues and the diverse nature of evidence that comes into play, such as evidence on health outcomes, behaviours or finances [1]. Health information systems research findings tend to be inconclusive, providing low- or very low-certainty evidence [39], partly due to these complexities and partly because of the use of observational—or quasi-experimental at best—study designs.

We have adhered to best practices when designing the research programme reported here. First, we have established the need for systematic reviews (WS2) to ensure that our research question could not have been answered with existing evidence [40, 41]. Second, we have used fieldwork (WS3) and innovative methods to design the intervention (HCD, WS4). Third, we have used a cluster-randomized controlled trial study design to address the research programme effectiveness question [39] (WS5), and fourth, we have explicitly taken into account contextual and policy environment issues [42] across the whole research programme (WS6). Furthermore, the experimental study design will ensure that PHISICC findings are sufficiently robust to be incorporated in future systematic reviews [43] and guidance development [44, 45].

We aimed at adding value to the HIS component of the PHISICC initiative through a series of options that we have taken over the life of the project. First, we have focused the whole project on decision-making, rather than on data per se and its technical aspects. In doing so, we prioritized decisions made by frontline health workers in their daily work routine, which are decisions at the patient of care or public health decisions. The paper-based intervention was created to support those decisions.

Second, the PHISICC paper tools intervention has been designed for all healthcare areas delivered in PHC services (i.e. antenatal care, delivery, vaccination, etc.), not siloed within one or two areas [46, 47]. This is important because, as we already realized, interactions between different healthcare areas unveil extraordinary new challenges and opportunities that remain unseen when research is limited to just one particular area. For example, looking at all healthcare areas, we realized the extraordinary volume of data that health workers have to handle and that the PHISICC interventions should tackle. We also accounted for the need to connect the PHISICC system with existing digital systems [48] operating at the district level.

Third, the push for digitalization of HIS worldwide, including in LMIC, is extraordinary and not without challenges [49]. Furthermore, the evidence supporting these efforts is very patchy and generally weak. However, early learnings from PHISICC suggest that there is no real conflict between paper and digital systems and that the future will likely require mixed systems combining the benefits of both paper-based and digital tools. With paper often being at the root of data collection, dismissing research on paper tools does not contribute to improving data quality and use at its source. Regardless of the format or technology of a data collection tool, the critical question is the degree to which it facilitates high-quality clinical decision-making through human-centred principles of usability and design. These principles are applicable to any HIS regardless of its technological support.

Fourth, we have taken an additional step forward in the intervention design by using a HCD approach which has put the users of the intervention at the centre of the intervention development. Design is a discipline that applies a specific mindset and skill set that can be both a complementary and innovative way to design tools and interventions in global health [50]. Design can help bring diverse disciplines together in a collaborative manner to unlock new opportunities for positive change in health systems. The result has been a set of tools with a visual language and clinical content that greatly contrast with the tabular forms of existing tools, as we have witnessed in the three countries (WS3) and elsewhere [51] and in those targeting higher levels of the system [52].

The main methodological limitation of our approach is related to the remoteness of the selected study areas. This was an explicit choice to contribute to making research in remote areas more visible. However, this has introduced additional challenges, mainly related to communication and transport of research teams and research participants, the means to carry out the design fieldwork, the frequency of trial monitoring visits and the turnover of staff in rural areas, among others. Accordingly, we have introduced several control mechanisms (i.e. frequent communications, trial monitoring) and relied on experienced research teams with a track record of research carried out in those areas where the most vulnerable live.

## Data Availability

Data will be made available once data collection and data analyses have been completed. Several publications are planned, and the data availability policies of the journals where these manuscripts will appear will be adhered to.

## Abbreviations

BMGF: Bill & Melinda Gates Foundation
HCD: Human-centred design
HIS: Health information systems
HMIS: Health management information systems
PHC: Primary healthcare
PHISICC: Paper-based Health Information System in Comprehensive Care
Swiss TPH: Swiss Tropical and Public Health Institute
TAG: Technical advisory group
WS: Workstream
